# An Experimental and Quantum Chemical Calculation Study on the Performance of Different Types of Ester Collectors

**DOI:** 10.3390/molecules30010147

**Published:** 2025-01-02

**Authors:** Di Wu, Jianhua Chen, Yuqiong Li

**Affiliations:** 1 School of Resources, Environment and Materials, Guangxi University, Nanning 530004, China; wdi0110@163.com; 2 State Key Laboratory of Featured Metal Materials and Life-Cycle Safety for Composite Structures, Guangxi University, Nanning 530004, China; 3 Guangxi Higher School Key Laboratory of Minerals Engineering, Guangxi University, Nanning 530004, China

**Keywords:** ester collectors, density functional theory, chalcopyrite, coordination interaction

## Abstract

Ester collectors have rapidly developed into the main flotation collectors for copper sulfide minerals since they were developed. In this study, the collecting performance of four collectors, O-isopropyl-N-ethyl thionocarbamate ester (IPETC), 3-pentyl xanthate acrylate ester (PXA), O-isobutyl-N-allyl-thionocarbamate (IBALTC), and O-isobutyl-N-isobutoxycarbonyl-thionocarbamate (IBIBCTC), was investigated through microflotation tests, microcalorimetric measurements, and quantum chemical calculations. The results of the microflotation tests show that IBALTC and IPETC have stronger collecting abilities than IBIBCTC and PXA; the order of collecting ability is IBALTC > IPETC > IBIBCTC > PXA. The microcalorimetry test also shows that the adsorption heat of the former two is higher. Quantum chemical calculations show the energy difference between the HOMOs of the collector and the LUMOs of minerals. The electrostatic potential extremum around S atom and the first ionization potential of IPETC and IBALTC are similar and were smaller than IBIBCTC and PXA, which shows that the collecting ability of the former two is similar and stronger than the latter two. Among the collectors, the S atom polarizability, electrophilic, and nucleophilic attack index of IBALTC are the largest, indicating that its electronic deformation capability and nucleophilic properties are the strongest, which results in the strongest coordination interaction with the copper ions in copper sulfide minerals and thus the highest collecting ability. The S atom polarizability, electrophilic, and nucleophilic attack index of PXA are the smallest, indicating that its electronic deformation capability and nucleophilicity are the weakest, and its collecting ability is the weakest. The coordination between collector and mineral surface was analyzed theoretically. The research results are of great help to the design and development of ester collectors.

## 1. Introduction

Copper is a non-ferrous metal closely related to human life. Due to its special properties, such as electrical conductivity and ductility, copper is widely used in fields like construction, electrical engineering, light industry, machinery manufacturing, and national defense industries [1]. Chalcopyrite (CuFeS_2_), the main source of copper, is the most widely used copper sulfide mineral, and foam flotation is the common technology used for its recovery [2]. Due to the excellent collecting ability of xanthate collectors, they are frequently used in the flotation of sulfide minerals. However, their poor selectivity has led to a search for alternative collectors that could replace xanthate collectors, which remains an ongoing research direction [3]. Since Harris and Fischback [4] applied for a process of commercial production of dialkylthionocarbamate collectors in 1954, ester collectors have rapidly become the main collectors for chalcopyrite flotation due to their good selectivity and being more stable than xanthates at low alkalinity [5].

Thionocarbamate (ROCSNR’) has the characteristics of strong selectivity and low dosage, with O-isopropyl-N-ethyl thionocarbamate (IPETC) being the most commonly used thionocarbamate collector [6]. In 1969, Falvey applied for a patent for S-allyl-N-alkyldithionocarbamate as a collector for chalcopyrite. Compared to O-isopropyl-N-ethyl thionocarbamate, S-allyl-N-alkyldithionocarbamate showed stronger collecting ability for chalcopyrite [7]. Ackerman et al. [8] studied the effect of N-alkyl and O-alkyl substituents on thionocarbamate. They believe that the introduction of isomeric groups at the N-alkyl position or the introduction of unsaturated groups in these two groups will lead to a decrease in the collecting ability. Liu et al. [9] used an ab initio method to investigate the effects of substituents on thionocarbamate. The results showed that O-isobutyl-N-ethoxycarbonyl-thionocarbamate (IBECTC) and O-isobutyl-N-acetyl-thionocarbamate (IBACTC) performed better as flotation collectors than O-isobutyl-N-allyl-thionocarbamate ester (IBALTC) and O-isobutyl-N-ethyl-thionocarbamate ester (IBETC).

Mkhonto et al. [10] studied O-butyl-Nethoxycarbonyl-thionocarbamate (BECTC), O-isobutyl-N-ethoxycarbonyl-thionocarbamate (IBECTC), O-butyl-Nbutoxycarbonyl-thionocarbamate (BBCTC), O-isobutyl-N-butoxycarbonyl-thionocarbamate (IBBCTC), and O-isobutylN-isobutoxycarbonyl-thionocarbamate (IBIBCTC) by density functional theory (DFT). They found that Oxycarbonyl-thionocarbamate preferentially adsorbs on Cu atoms rather than Fe atoms. The adsorption energy followed a trend similar to mineral recovery, with the following order: BBCTC > IBIBCTC > BECTC > IBBCTC > IBECTC.

Xanthate derivatives, such as xanthate ester (ROCSSR’), were also used in chalcopyrite flotation. Huang et al. [11] studied the derivative of S-hydroxyethyl-O-isobutyl xanthate (HEIBX) and found it has good selectivity in chalcopyrite flotation. Xiao et al. [12] proved the selectivity of O-isopropyl-S-[2-(hydroxyimino)propyl] dithiocarbonate (IPXPO). Liu et al. [13] studied S-[(2-hydroxyamino)-2-oxoethyl 1]-O-octyl-dithiocarbonate (HAOODE) and further improved chalcopyrite recovery by adding hydroxamic acid groups.

Since the 1970s, density functional theory (DFT) has been widely used in the calculation of solid state physics. Computational chemistry was recognized as equally important as experimental chemistry in studying chemical reactions and physical and chemical properties in chemical systems [14,15,16]. DFT was also extensively used to study the interfacial chemical reactions between collectors and minerals to understand flotation mechanisms [17,18]. This study combines microflotation experiments and microcalorimetry with DFT calculations to investigate the collecting abilities of four ester collectors for chalcopyrite. The frontier orbital, atomic charge, electrostatic potential, polarizability, first ionization potential, and electrophilic nucleophilic index of different collectors were calculated, and the structure–performance relationship of ester collectors was established. The results are significant for the development of ester collectors.

## 2. Results and Discussion

### 2.1. Microflotation Tests Results

**Figure 1:**
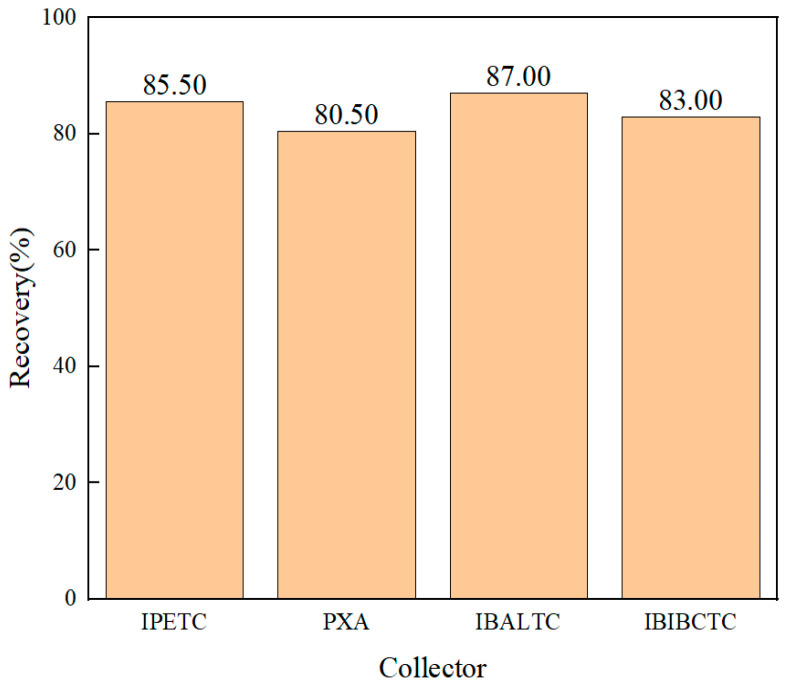
Microflotation recoveries of chalcopyrite using IPETC, PXA, IBALTC, and IBIBCTC collectors.

The flotation recovery rate of chalcopyrite as a function of collector type at a collector concentration of 2 × 10^−5^ mol/L and pH7 is shown in Figure 1. The recovery rate reflects the flotation performance of these collectors. The recoveries of IPETC and IBALTC were 85.50% and 87.00%, respectively, and the difference between them was small and significantly higher than those of PXA and IBIBCTC. This indicates that their collecting ability for chalcopyrite is stronger than that of PXA and IBIBCTC. The recovery rate of IBIBCTC is 83.00%, higher than that of PXA at 80.50%, suggesting that PXA has the weakest collecting ability.

### 2.2. Microcalorimetric Measurement Results

**Figure 2:**
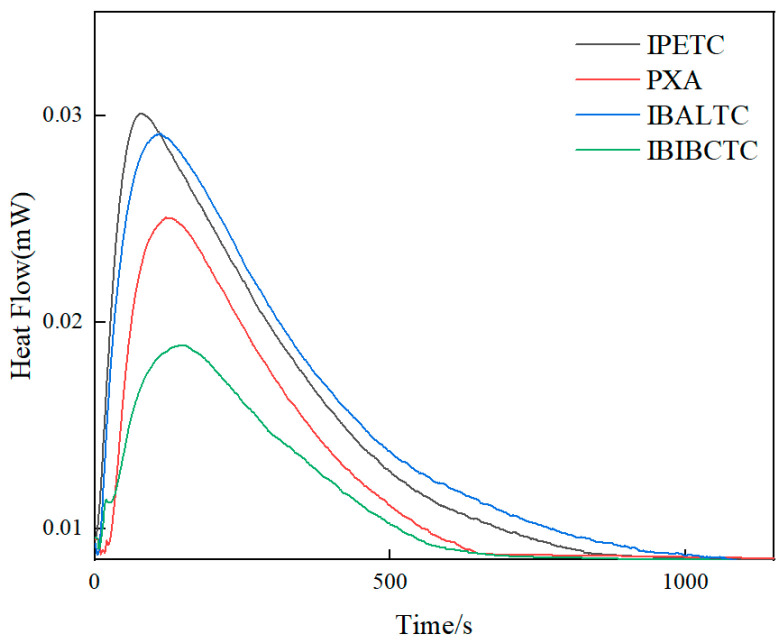
Heat flow of different collectors on chalcopyrite surface.

For a chemical reaction under isothermal and isobaric conditions, the change in enthalpy is proportional to the progress of the reaction, and the rate of change in enthalpy with time is proportional to the reaction rate. The calorimeter can accurately measure the heat transformation during the reaction process, providing the thermodynamic change curve, which is significant for elucidating the thermal effects of interfacial reactions. Microcalorimetry was used in studies on mineral surface activation and adsorption mechanisms [19,20]. The microcalorimetric thermodynamic curves of different collectors on the chalcopyrite surface are shown in Figure 2.

The total thermal effect of the change process is the integral area under the curve.

(1) $$\int_{t0}^{t}\frac{dH_{t}}{dt}$$

The reaction heat Q is shown in Table 1. The reaction heats for all the collectors are positive, indicating that the reactions are exothermic. The maximum reaction heat is 349.34 mJ for IBALTC, followed by IPETC with a reaction heat of 218.12 mJ. The reaction heats for PXA and IBIBCTC are lower than the former two, at 165.08 mJ and 132.54 mJ, respectively. This suggests that IBALTC and IPETC adsorb more strongly on the mineral surface than PXA and IBIBCTC. The reaction rate constant k and the reaction order n can be determined using the following equation:

(2) $$\ln[\frac{1}{H_{\infty }}\frac{dH_{i}}{dt}]\;=\mathrm{lnk}+\mathrm{nln}\;[1-\frac{H_{i}}{H_{\infty }}]$$

The reaction rate constant k reflects the speed of the adsorption process. The k values for the four collectors are 3.70 × 10^−3^ s^−1^, 2.93 × 10^−3^ s^−1^, 1.66 × 10^−3^ s^−1^, and 3.45 × 10^−3^ s^−1^, indicating that IPETC has the fastest reaction rate, followed by IBIBCTC and PXA, with IBALTC having the slowest reaction rate. The reaction orders n for the four collectors are 0.86, 0.64, 0.97, and 0.70, indicating that all reactions are pseudo first order reactions.

### 2.3. Molecular Structure and Properties of Collectors

#### 2.3.1. Difference in C=S Bond in Collectors

**Figure 3:**
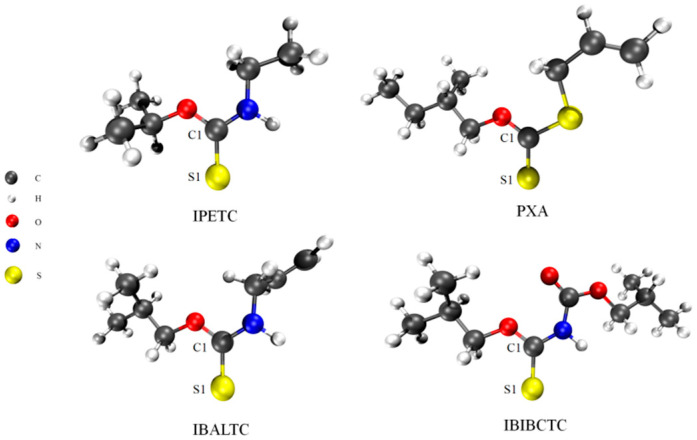
Geometry of collectors’ molecular structures after optimization.

In order to explore the relationship between the properties of ester-based collectors and their flotation performance, the molecular configurations of the four collectors were optimized at the B3LYP/6-311G(d,p) level (as shown in Figure 3). The frequency of the optimized results is calculated; all frequencies are positive, and the imaginary frequency is 0. The optimized geometric parameters are presented in Table 2. The detailed geometric structure is shown in Table S1.

Bond order can measure the relative strength of the covalent bonds. The larger the bond order, the shorter the bond length, and the stronger the covalency. This section mainly analyzes the bond length and Mayer bond order of the C=S bond. Mayer bond order is calculated by Multiwfn (3.8 version). The C=S bond lengths and bond orders for IPETC and IBALTC are similar, with bond lengths of 1.701 Å and 1.697 Å, and bond orders of 1.454 and 1.463, respectively. The C=S bond in IBIBCTC is slightly shorter than the former two, but the bond order is larger (1.602). The C=S bond in PXA is the shortest (1.664 Å) and has the largest bond order (1.611). These results suggest that the covalency of the C=S bond in thionocarbamates is weaker than that in xanthate ester.

#### 2.3.2. Frontier Orbital, Atomic Charge, and Electrostatic Potential of Molecules

Fukui [21] proposed that many properties of a molecule are primarily determined by the frontier molecular orbital (FMO) of the molecule. The highest occupied molecular orbital (HOMO) of one molecule participating in the reaction must match the symmetry of the lowest unoccupied molecular orbital (LUMO) of the other molecule, and their energy levels must be close to each other (<6 eV). The smaller the absolute value of energy difference (ΔE) between the HOMO of one reactant and the LUMO of the other, the more favorable the interaction between the two is. The shape and composition of the frontier molecular orbital of the collector can intuitively show the atoms that contribute the most, which is the active position of the reaction and the bonding atom of the collector.

**Figure 4:**
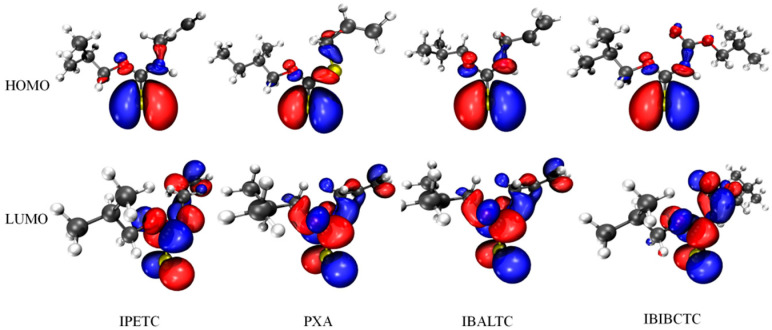
Frontier orbital configurations of collector molecules.

It can be clearly noted from Figure 4 that the electron cloud of the HOMO is mainly located on the S atom, while the electron cloud of the LUMO is primarily located on the S atom and the C atom of the C=S bond, which indicates that the bonding atom that forms a normal covalent bond with the sulfide mineral is the S atom, and the S atom is the reactive center. Additionally, the shape of the frontier orbitals shows that the HOMO is a bonding π orbital, while the LUMO is an empty π antibonding orbital.

To assess the collecting ability of the collectors based on the frontier molecular orbital energy, the HOMO and the LUMO of each collector were calculated. The orbital energy differences between the different collector molecules and chalcopyrite were then calculated using Formulas (3) and (4) [22,23,24].

(3) $$\Delta E_{1}=|E_{HOMO}^{Collector}-E_{LUMO}^{CuFeS2}|$$

(4) $$\Delta E_{2}=|E_{HOMO}^{CuFeS2}-E_{LUMO}^{Collector}|$$

Chen and Wang [25] calculated the HOMO and LUMO energies of chalcopyrite (CuFeS_2_) using Material Studio (4.2 version) software. The RPBE gradient correction function under the generalized gradient approximation (GGA) is adopted. The HOMO and LUMO energies of chalcopyrite and the collector are listed in Table 3, and the values are calculated. ΔE1 is much smaller than ΔE2, indicating that the interaction between the collector and the mineral mainly comes from the HOMO of the collector and the LUMO of the mineral. The collector provides electrons from the HOMO of the central S atom to form a covalent bond with the copper ion. The ΔE2 value is larger but less than 6 eV, indicating that the LUMOs of the four collectors can also interact with the HOMOs of chalcopyrite to form a π-back bond. The order of the four ΔE1 values is as follows, with the former two being close to each other (1.46 eV and 1.49 eV), and the last two also being close (1.81 eV and 1.86 eV): IPETC < IBALTC < PXA < IBIBCTC. This indicates that the HOMO of IPETC and IBALTC interacts more strongly with the LUMO of chalcopyrite, which is consistent with the stronger collecting abilities of IPETC and IBALTC to collect chalcopyrite. It is worth noting that although the ΔE1 of PXA and IBIBCTC is larger, the ΔE2 is smaller, suggesting that their π-back bonds formed with chalcopyrite may be stronger, which could contribute to their collecting abilities for chalcopyrite.

The Restrained ElectroStatic Potential (RESP) charges, proposed by Kollman et al. in 1993 [26], are one of the most commonly used methods for calculating atomic charges in molecular dynamics simulations. The charges of the central S atom of the four collectors were calculated and are listed in Table 3, with values of −0.61 e^−^, −0.43 e^−^, −0.58 e^−^, and −0.46 e^−^. The C=S bond structures of IPETC and IBALTC are similar, and the charges on the sulfur atoms are also close, with significantly more negative charge compared to PXA and IBIBCTC. The electron-withdrawing oxycarbonyl group connected to the N atom of IBIBCTC reduces the charge on the central S atom. In PXA, the allyl group has electron-withdrawing properties, which also leads to a decrease in the charge on the central S atom.

**Figure 5:**
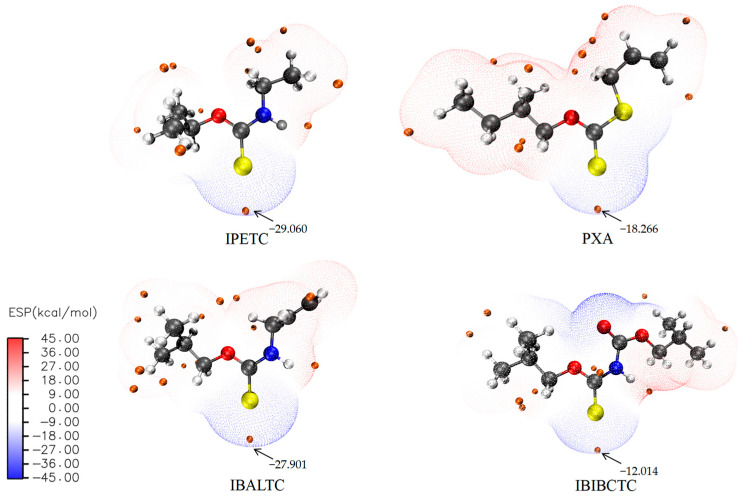
Electrostatic potential surface of collectors.

An electrostatic potential (ESP) diagram provides an intuitive view of the area where the collector is easily able to obtain or lose electrons and the active site of the reaction. As shown in Figure 5, the blue regions represent negative electrostatic potential values, indicating that electrons in these regions are at higher energy levels, making them more likely to undergo electrophilic attack and donate electrons. The red regions represent positive electrostatic potential values, indicating that it is prone to nucleophilic attacks and able to accept electrons. The orange dots represent the extreme points. From Figure 5, it can be seen that the region with the lowest negative electrostatic potential is the same as the position of the collector HOMO electron cloud surrounding the central S atom. This suggests that the S atom is the reaction center and is prone to electrophilic attack. The electrostatic potential extreme value of IPETC and IBALTC are similar and relatively lower (−29.060 kcal/mol and −27.901 kcal/mol) hence the ability to interact with the mineral surface will be stronger. The electrostatic potential extreme value of PXA and IBIBCTC are −18.266 kcal/mol and −12.014 kcal/mol, which are higher than the former two collectors, suggesting weaker interactions with the mineral surface than the former two collectors.

#### 2.3.3. Polarizability, First Ionization Potential, and Electrophilic Nucleophile Index of Collectors

The polarizability represents the degree of delocalization of electrons in the molecules in the electric field. It is a measure of the ability of the electron cloud to deform under the influence of an external electric field. The larger the polarizability, the easier it is to delocalize its electrons and the stronger its ability is to form covalent coordination with the mineral surface. Previous studies have shown that the S atom is the reaction center, so the polarizability of the sulfur atom is calculated.

As shown in Table 4, the polarizability of the S atom in IBALTC is the highest (20.54 a.u.), indicating that the delocalization of the S atom in IBALTC is stronger. The electrons in the outermost p orbitals of the S atom are more easily delocalized into the outermost vacant orbitals of the copper ion, resulting in a stronger covalent bond. This is consistent with the highest flotation recovery rate of chalcopyrite for IBALTC. The polarizabilities of the S atoms in IPETC and IBIBCTC are similar (19.55 a.u. and 19.42 a.u.), which are lower than that of IBALTC, indicating weaker electron delocalization compared to IBALTC. Consequently, their ability to form covalent coordination with the mineral surface and their collecting ability for chalcopyrite are weaker than that of IBALTC. PXA has the lowest polarizability of the S atom (18.93 a.u.), meaning it has the weakest electron delocalization and the weakest ability to form covalent coordination with the chalcopyrite surface, resulting in the weakest collecting ability for chalcopyrite.

The first ionization potential refers to the energy change when a neutral atom or molecule loses one electron to become a +1 valence ion. The lower the value, the easier it is for the molecule to lose an electron. The +1 valence states of the four collected molecules were geometrically optimized, and their energies were calculated. The calculation parameters were the same as those in the ground state. The result is subtracted from the energy in the ground state to obtain the first ionization potential. As shown in Table 4, the first ionization potentials of IBALTC and IPETC are 6.08 eV and 6.19 eV, which are close and lower than those of PXA and IBIBCTC. This indicates that IBALTC and IPETC are more likely to lose electrons, showing higher reactivities and stronger collecting abilities for the minerals. The first ionization potentials of PXA and IBIBCTC are 6.42 eV and 6.59 eV, meaning their abilities to lose electrons is weaker than IBALTC and IPETC, and their reactivities are worse, so their abilities to collect minerals are weaker than IBALTC and IPETC.

The Fukui function is a function that describes the reactivity of molecules. It was proposed by Parr and Yang [27]. The indices $f^{-}$ and $f^{+}$ represent the electrophilic attack index and the nucleophilic attack index. It represents the degree of difficulty in losing or obtaining an electron when the system is attacked by an electrophilic reagent or a nucleophilic reagent. The larger the value, the easier it is to lose or gain an electron; $f^{2}$ is the difference between $f^{+}$ and $f^{-}$. If $f^{2}\;$> 0, atom is electrophilic; if $f^{2}\;$< 0, the atom is nucleophilic.

The electrophilic and nucleophilic attack indices of the central S atom were calculated, and the results are shown in Table 5. All $f^{2}$ are negative, indicating that the central S atom is nucleophilic. The $f^{2}$ of IBALTC, IPETC, and IBIBCTC are very close, and their negative values are significantly larger than PXA, which indicates that the S atom in PXA has the smallest nucleophilicity. The nucleophilicity of the S atoms in the former three is close. Moreover, we conducted further analyses of the $f^{-}$ and $f^{+}$ values. The $f^{+}$ of IBALTC, IPETC, IBIBCTC, and PXA are all close, ranging from 0.34 to 0.38, while the $f^{-}$ value of PXA is about 0.40, which is noticeably lower than the other three (around 0.65). This suggests that the lower nucleophilicity of PXA was caused by its lower electrophilic attack index but not nucleophilic attack index. From these results, it can be concluded that the nucleophilicity of the S atom has a significant influence on its collecting ability, with greater nucleophilicity corresponding to a stronger collecting ability for chalcopyrite.

#### 2.3.4. Coordination Model of Collector and Chalcopyrite Surface

**Figure 6:**
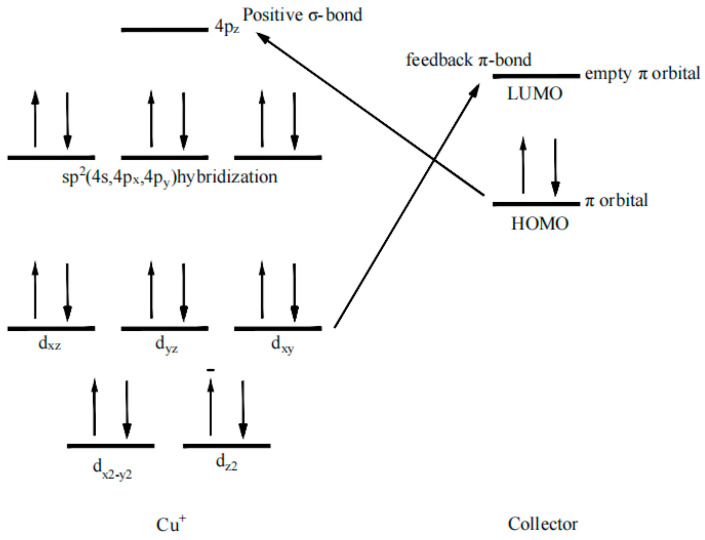
Coordination model between surface Cu^+^ and collectors.

Chen [28] proposed that the interaction between reagent molecules and mineral surfaces is mainly governed by structural matching and orbital matching. The orbital matching analysis of copper ions and collectors was performed, as shown in Figure 6. From the shape of the collector frontier orbitals, it can be seen that the HOMOs of the four collectors are π orbitals, and the LUMOs are empty π orbitals. On the surface of chalcopyrite, the Cu ion is in the +1 valence [29], the d orbital of Cu^+^ is 3d^10^, and the outer valence electrons 4s and 4p are empty orbitals. Therefore, for the three-coordinate Cu^+^, one 4s orbital and two 4p orbitals (4p_x_, 4p_y_) hybridize to form sp^2^ hybridized orbitals, which interact with three sulfur atoms to form a planar triangular structure. The 4p_z_ orbital remains vacant and can accept the electron pairs from the collector molecules, forming a coordination interaction, and becoming a four-coordinate structure with sp^3^ hybridized. Meanwhile, the LUMO of the collector can accept electron pairs from Cu^+^ to form a π-back bond.

## 3. Experimental and Computational Methods

### 3.1. Microflotation Tests

**Figure 7:**
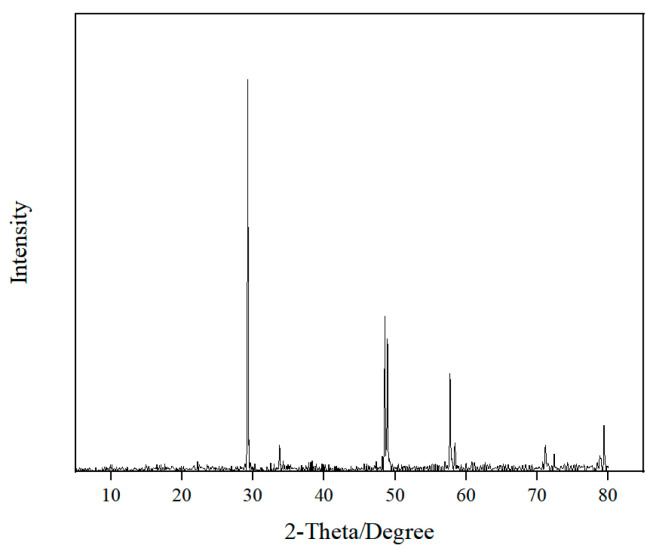
X-ray diffraction spectra of chalcopyrite.

The collecting ability of four collectors for chalcopyrite was investigated through microflotation tests. The chalcopyrite samples were obtained from a mine in Yunnan, China. The mineral composition and chemical composition of the chalcopyrite were determined by X-ray diffraction (XRD) and X-ray fluorescence spectroscopy (XRF), as shown in Figure 7 and Table 6. The purity of the chalcopyrite obtained was approximately 93.03%.

The chalcopyrite was crushed and drily ground to obtain a product with a particle size between −74 + 45 μm, which was used for the microflotation tests. The mass of the mineral feed was 2.0 g, and ultrapure water was employed for the flotation process. Prior to flotation, the mineral was cleaned by ultrasound for 3 min. Flotation experiments were conducted on an XFGCII flotation machine with a 60 mL flotation cell. The stirring speed was controlled at 1740 r/min. The collector and frother (MIBC) were separately added to the slurry and stirred for 2 min each. The flotation time was set to 2 min, and the concentrates were dried in an oven and weighed. The recovery rate was calculated from Equation (1) as follows:

(5) $$Recovery\;(\%)=\frac{Mass\;of\;mineral\;in\;concentrate}{Mass\;of\;mineral\;in\;feed}$$

### 3.2. Microcalorimetric Measurement

The chalcopyrite sample used for microcalorimetric measurements was the same as the one used in the microflotation experiments. The concentration of the collector was 1 × 10^−4^ mol/L, and the pH was 7. Measurements were conducted at 23 °C using an RD496-2000 calorimeter (Mianyang, China). This calorimeter has a baseline stability of ±0.12 μW, a temperature stability of ±0.0001 °C, a heating rate of 0.01–2.00 °C/min, and a resolution of 0.1 mW. For each measurement, 0.20 g of the mineral sample was placed in the lower chamber of the reaction cell and reference cell. The lower part of the reaction cell was sealed with a polytetrafluoroethylene sealing ring covered with an aluminum membrane. Then, 1.00 mL of the flotation collector solution was added to the upper chamber of the reaction cell. Each measurement was performed after the calorimeter baseline had fully stabilized. A movable needle was used to puncture the upper glass partition, allowing the collector solution to react with the mineral particles. Finally, the obtained data were analyzed using SETSOFT (2000 version) software.

### 3.3. Computational Methods

The molecules of the input file were visualized using Gauss View 6, and quantum chemical calculations were performed using the Gaussian 16 software package. Density functional theory (DFT) was employed with the B3LYP/6-311G(d,p) method [30], and the solvent effect of water was simulated using the Integral Equation Formalism Polarizable Continuum Model (IEFPCM) under the Self-Consistent Reaction Field (SCRF) approach [31,32]. The B3LYP functional is the most widely used functional in the field of quantum chemical calculations since its appearance in 1994. It is also the most frequently used functional in the study of flotation collectors [33,34,35] as 6-311G (d, p) is a common basis set, which is often used in the geometric optimization of collector molecules [36,37,38]. The results were analyzed and visualized using Multiwfn and VMD [39,40,41]. Geometric optimization was carried out for four collectors, O-isopropyl-N-ethyl thionocarbamate (IPETC), 3-pentyl xanthate acrylate ester (PXA), O-isobutyl-N-allyl-thionocarbamate (IBALTC), and O-isobutyl-N-isobutoxycarbonyl-thionocarbamate (IBIBCTC), to obtain their stable molecular structures, energies, and charge distributions.

## 4. Conclusions

This study systematically investigates the collecting ability of four ester collectors through microfloation tests, microcalorimetric measurements, and computational simulations, and the coordination interaction model between the collector and the mineral surface was established.

According to the microfloation test results, the recovery rates of IPETC and IBALTC are similar and higher than those of PXA and IBIBCTC, indicating that their collecting ability is stronger than PXA and IBIBCTC. Microcalorimetric measurements show that the reaction heats of IPETC and IBALTC are higher than those of PXA and IBIBCTC, suggesting that the adsorptions of IPETC and IBALTC on the chalcopyrite surface are stronger than those of PXA and IBIBCTC. DFT calculation results show that the HOMOs of the collectors and the negative regions of electrostatic potential are concentrated around the central S atoms, indicating that the S atoms of the collectors are the reactive centers. The interaction between the collectors and the mineral surface frontier molecular orbitals mainly comes from the positive covalent interaction between the electrons on the HOMO of the collector and the LUMO of the mineral. Although the energy difference between the LUMO of the collectors and the HOMO of chalcopyrite is larger, it is still less than 6 eV, indicating that the LUMO of the collectors can accept electron pairs from Cu+ to form a π-back bond. The energy difference between the HOMO of IPETC and IBALTC and the LUMO of chalcopyrite, the electrostatic potential extrema, and the first ionization potentials are similar and smaller than those of PXA and IBIBCTC, indicating that their collecting abilities are stronger than PXA and IBIBCTC. The polarization rate and electrophilic attack index of IBALTC, IPETC, and IBIBCTC are much larger than those of PXA, suggesting that PXA has the weakest collecting ability. IBALTC has the highest polarization rate and electrophilic attack index, indicating that its collecting ability is stronger than the other three. The theoretical results are consistent with the experimental values.

## Figures and Tables

**Table 1:** Thermal kinetic parameters of different collectors adsorbed on chalcopyrite surface.

Collector	Q (mJ)	k (×10^−3^ s^−1^)	n
IPETC	218.12	3.70	0.86
PXA	165.08	2.93	0.64
IBALTC	349.34	1.66	0.97
IBIBCTC	132.54	3.45	0.70

**Table 2:** Geometric parameters of collectors after optimization.

Collector	C1=S1 Bond Length (Å)	C1=S1 Mayer Bond Order
IPETC	1.701	1.454
PXA	1.664	1.611
IBALTC	1.697	1.463
IBIBCTC	1.674	1.602

**Table 3:** HOMO and LUMO energies for the collectors and chalcopyrite and central S atom RESP atomic charges.

Collector	HOMO and LUMO Energies (eV)		$∆E_{1}$(eV)	$∆E_{2}$(eV)	RESP Atomic Charges (e^−^)
	HOMO	LUMO			
IPETC	−6.34	−0.51	1.46	5.11	−0.61
PXA	−6.69	−1.65	1.81	3.97	−0.43
IBALTC	−6.37	−0.68	1.49	4.94	−0.58
IBIBCTC	−6.74	−1.58	1.86	4.04	−0.46
CuFeS_2_	−5.62	−4.88	—	—	—
Collector	HOMO and LUMO energies (eV)		$∆E_{1}$(eV)	$∆E_{2}$(eV)	RESP atomic charges (e^−^)

**Table 4:** Polarizability and first ionization potential of S atom.

Collector	Polarizability of S Atom (a.u.)	First Ionization Potential of S Atom (eV)
IPETC	19.55	6.19
PXA	18.93	6.42
IBALTC	20.54	6.08
IBIBCTC	19.42	6.59

**Table 5:** Electrophilic nucleophile index of S atom.

Collector	$\mathrm{Electrophilic}\;\mathrm{Attack}\;\mathrm{Index}\;f^{-}$	$\mathrm{Nucleophilic}\;\mathrm{Attack}\;\mathrm{Index}\;f^{+}$	$f^{2}=f^{+}-f^{-}$
IPETC	0.65	0.34	−0.31
PXA	0.40	0.38	−0.02
IBALTC	0.67	0.34	−0.33
IBIBCTC	0.64	0.35	−0.29

**Table 6:** Chemical composition of chalcopyrite samples (wt%).

Element	Cu	Fe	S
chalcopyrite	32.15	32.97	33.15

## Data Availability

The data presented in this study can be obtained by contacting the corresponding author.

## Supplementary Materials

The following supporting information can be downloaded at: https://www.mdpi.com/article/10.3390/molecules30010147/s1. Figure S1. Geometry of collectors’ molecular structures after optimization. Table S1. Geometric parameters of collectors after optimization. References [3,9,10,42].
