# Supplementary material for: An Experimental and Quantum Chemical Calculation Study on the Performance of Different Types of Ester Collectors
Source: Molecules. 2025 Jan 2;30(1):147. doi: 10.3390/molecules30010147 (PMC11721024; doi:10.3390/molecules30010147)
Supplement: Supplementary file 1 [file molecules-30-00147-s001.zip › molecules-3387785-supplementary.pdf]

Supplementary File

For

An Experimental and Quantum Chemical Calculation Study on the Performance of Different Types of Ester Collectors

Di Wu 1, Jianhua Chen [1,2,3]*, Yuqiong Li [1,2,3]*

[1] School of Resources, Environment and Materials, Guangxi University, Nanning 530004, China; wdi0110@163.com
[2] State Key Laboratory of Featured Metal Materials and Life-Cycle Safety for Composite Structures, Guangxi University, Nanning 530004, China
[3] Guangxi Higher School Key Laboratory of Minerals Engineering, Guangxi University, Nanning 530004, China

The molecular configurations of the four collectors were optimized at the B3LYP/6-311G(d,p) level (as shown in Figure S1). The frequency of the optimized results is calculated; all frequencies are positive, and the imaginary frequency is 0. The optimized geometric parameters are presented in Table S1.

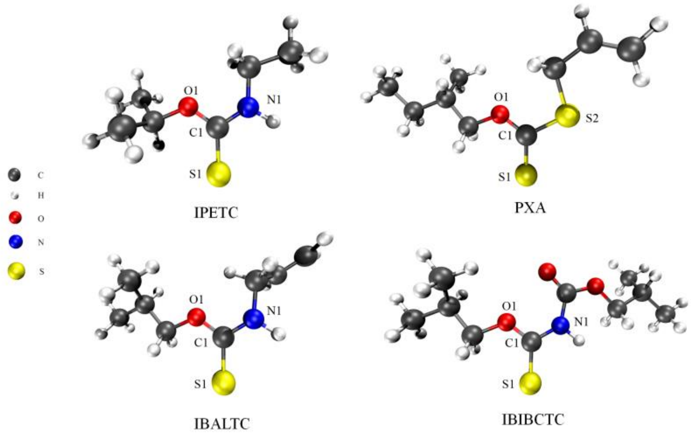

Figure S1. Geometry of collectors' molecular structures after optimization.

Table S1. Geometric parameters of collectors after optimization.

bond	IPETC	PXA	IBALTC	IBIBCTC
C1-S1	1.701	1.664	1.697	1.674
C1-O1	1.329	1.317	1.329	1.309
C1-N1	1.335	—	1.338	1.381
C1-S2	—	1.760	—	—

The C1-S1 bond length, C1-O1 bond length and C1-N1 bond length of ethyl xanthate obtained by Mkhonto [3] et al. are 1.657 Å, 1.366 Å, and 1.353 Å, and the error is less than 3 %.

The C1-S1 bond length obtained by Claes [42] et al. was 1.636Å, and the C1-S2 bond length

was 1.761Å. Compared to PXA, the error was less than 2 %.

According to Liu et al. [9], the structural parameters of IBALTC are a C1-S1 bond length of 1.671 Å, a C1-O1 bond length of 1.339 Å, and a C1-N1 bond length of 1.349 Å. The errors are less than 2 %.

According to the study of Mkhonto [10] et al., the structural parameters of IBIBCTC are a C1-S1 bond length of 1.651 Å, a C1-O1 bond length of 1.330 Å, and aC1-N1 bond length of 1.386 Å, with a less than 1.5 % error.

The above results show that the geometric optimization of the four collector molecules is reliable.

Reference

3. Mkhonto, P.P.; Zhang, X.R.; Lu, L.; Xiong, W.; Zhu, Y.G.; Han, L.; Ngoepe, P.E. Adsorption mechanisms and effects of thiocarbamate collectors in the separation of chalcopyrite from pyrite minerals: DFT and experimental studies. Minerals Engineering, 2022, 176. https://doi.org/10.1016/j.mineng.2021.107318.

42. Claes, L.; François, J.P.; Deleuze, M.S. Theoretical study of the internal elimination reactions of xanthate precursors. Journal of Computational Chemistry, 2003, 24, 2023-2031. https://doi.org/10.1002/jcc.10358

9. Liu, G.Y.; Zhong, H.; Dai, T.G.; Xia, L.Y. Investigation of the effect of N-substituents on performance of thionocarbamates as selective collectors for copper sulfides by ab initio calculations. Minerals Engineering, 2008, 21, 1050-1054.https://doi.org/10.1016/j.mineng.2008.04.017.

10. Mkhonto, P.P.; Zhang, X.R.; Lu, L.;Zhu, Y.G.; Han, L.; Ngoepe, P.E. Unravelling the performance of oxycarbonyl-thiocarbamate collectors on chalcopyrite using first-principles calculations and micro-flotation recoveries. Applied Surface Science, 2021, 563. https://doi.org/10.1016/j.apsusc.2021.150332.
